# Platelet polyphosphate and energy metabolism in professional male athletes (soccer players): A cross‐sectional pilot study

**DOI:** 10.14814/phy2.15409

**Published:** 2022-08-03

**Authors:** Takashi Ushiki, Tomoharu Mochizuki, Katsuya Suzuki, Masami Kamimura, Hajime Ishiguro, Satoshi Watanabe, Go Omori, Noriaki Yamamoto, Tomoyuki Kawase

**Affiliations:** ^1^ Division of Hematology and Oncology, Graduate School of Health Sciences Niigata University Niigata Japan; ^2^ Department of Transfusion Medicine, Cell Therapy and Regenerative Medicine Niigata University Medical and Dental Hospital Niigata Japan; ^3^ Department of Hematology, Endocrinology and Metabolism, Faculty of Medicine Niigata University Niigata Japan; ^4^ Department of Orthopaedic Surgery, Graduate School of Medical and Dental Sciences Niigata University Niigata Japan; ^5^ Department of Orthopaedic Surgery Niigata Medical Center Niigata Japan; ^6^ Department of Health and Sports, Faculty of Health Sciences Niigata University of Health and Welfare Niigata Japan; ^7^ Department of Orthopaedic Surgery Niigata Rehabilitation Hospital Niigata Japan; ^8^ Division of Oral Bioengineering, Graduate School of Medical and Dental Sciences Niigata University Niigata Japan

**Keywords:** athletes, basal metabolic rate, NADH, platelets, polyphosphates

## Abstract

Human platelet polyphosphate (polyP) is a multifunctional molecule; however, its functions are not yet fully understood. A recent study demonstrated that similar to skeletal muscle, polyP is involved in energy metabolism in platelets, which suggests that well‐trained athletes may exhibit elevated platelet polyP levels for energy storage. To test this hypothesis, we quantified platelet polyP along with NADH, a component involved in ATP production in non‐trained and well‐trained male Japanese participants of the same generation. Washed platelets were prepared from the venous blood of young, healthy, non‐athletes, and professional soccer players (pro‐athletes). NADH and polyP levels were spectrophotometrically determined using tetrazolium reduction and fluorometrically determined using 4′,6‐diamidino‐2‐phenylindole at the excitation/emission wavelengths of 425/525 nm. Body weight and impedances were measured simultaneously. Statistical analyses were performed using the Mann–Whitney *U* test and Spearman correlation coefficient. Although basal metabolic rate levels were significantly higher, platelet polyP levels were significantly lower in pro‐athletes than in that in non‐athletes. No significant differences were detected in other body compositions or platelet indices between the two groups. The pro‐athlete group showed a moderate, nearly significant correlation (*R* = 0.439; *p* = 0.0512) between platelet polyP and NADH levels. Taken together with the weak correlation data between polyP and body mass index, it is suggested that platelet polyP levels may be influenced by platelet and body energy metabolic activity. Further biochemical studies are needed to elucidate this mechanism.

## Graphical abstract

Although polyphosphate (polyP) has recently been accepted as a metabolite of energy metabolism, its metabolism and function remain poorly explored in human platelets. It could be hypothesized that well‐trained professional athletes who generally exhibit higher basal metabolic rate than non‐athletes may also exhibit elevated levels of platelet polyP for energy storage. This cross‐sectional study highlights that against our hypothesis, platelet polyP levels are significantly lower in elite athletes than in non‐athletes.

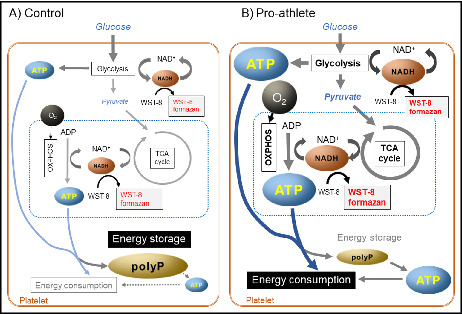

## 1 INTRODUCTION

Inorganic polyphosphate (polyP) is a negatively charged multifunctional polymer composed of multiple orthophosphates bound by high‐energy phosphoanhydride bonds (Hambardikar et al., 2022). Unlike those in bacteria, polyP synthesis nor its physiological functions are yet fully understood in mammalian cells (Achbergerová & Nahálka, 2011). However, recent studies have revealed that polyP is involved in the following biological processes: blood coagulation, immune responses, proliferation, mitochondrial ion transport, and respiration chain activity (Pavlov et al., 2010; Simbulan‐Rosenthal et al., 2019). Since the latter two functions are involved in energy metabolism, it has been postulated that polyP may function as an energy source in mammalian cells (McIntyre & Solesio, 2021; Müller et al., 2017), similar to that in bacteria (Pavlov et al., 2010).

Platelets are anuclear cell fragments arising from bone marrow megakaryocytes and play a pivotal role in both physiological hemostasis and thrombosis (Garcia‐Souza & Oliveira, 2014; Ravera & Panfoli, 2019). As in other nuclear cells, platelets contain functional mitochondria to generate energy and drive cellular actions. In contrast to major nucleated cells containing hundreds of functional mitochondria, platelets are known to be metabolically active, based on the substantially higher levels of ATP turnover, despite containing only 5–8 mitochondria per cell (Melchinger et al., 2019). In platelets, ATP is generated via two major processes: glycolysis and oxidative phosphorylation (OXPHOS). In a classic study (Kilkson et al., 1984), approximately 85% of the energy generation in resting platelets was mediated via OXPHOS, while glycolysis compensated for a shortage of energy in the activated state (Aibibula et al., 2018; Garcia‐Souza & Oliveira, 2014). To date, this concept has been considered a fundamental theory. However, a recent study released by Panfoli's group has highlighted conflicting theories against the classic one (Ravera et al., 2018): platelets could possess an extramitochondrial energy production system to respond to various agonist stimuli, necessitating a considerable amount of energy (Ravera & Panfoli, 2019). This was also supported by a more recent study focused on ultramarathon running. The authors reported that both OXPHOS and glycolysis produce platelet energy in the resting state and that only OXPHOS is responsible for platelet energy in the activated state (Hoppel et al., 2021). However, this finding could be attributed to possible metabolic differences between non‐athletes and well‐trained elite athletes.

In athletes, skeletal muscles are trained to maximize physical performance by developing (and facilitating) the turnover of muscle fiber and the energy supply system. In contrast to platelets, skeletal muscle cells meet high ATP requirements for muscle contraction mainly via oxygen‐dependent mechanisms in several hundred mitochondria per cell, potentially in cooperation with additional energy generation systems (Balberova et al., 2021; Greenhaff et al., 2002; Knuiman et al., 2015). Despite the substantial difference in energy demand, recent studies have demonstrated that mitochondrial function in circulating blood cells can reflect mitochondrial energetics of peripheral tissue, including skeletal muscle (Braganza et al., 2019; Tyrrell et al., 2016, 2017; Winnica et al., 2019). If accurate, this finding has several clinical merits. For example, highly expensive and invasive skeletal muscle biopsies can be avoided (Braganza et al., 2019). Furthermore, it may be helpful for the diagnosis of early sarcopenia in older adults (Braganza et al., 2019), as well as for determining the prognosis of muscle regenerative therapy using platelet‐rich plasma in young athletes (Simbulan‐Rosenthal et al., 2019).

**FIGURE 1:**
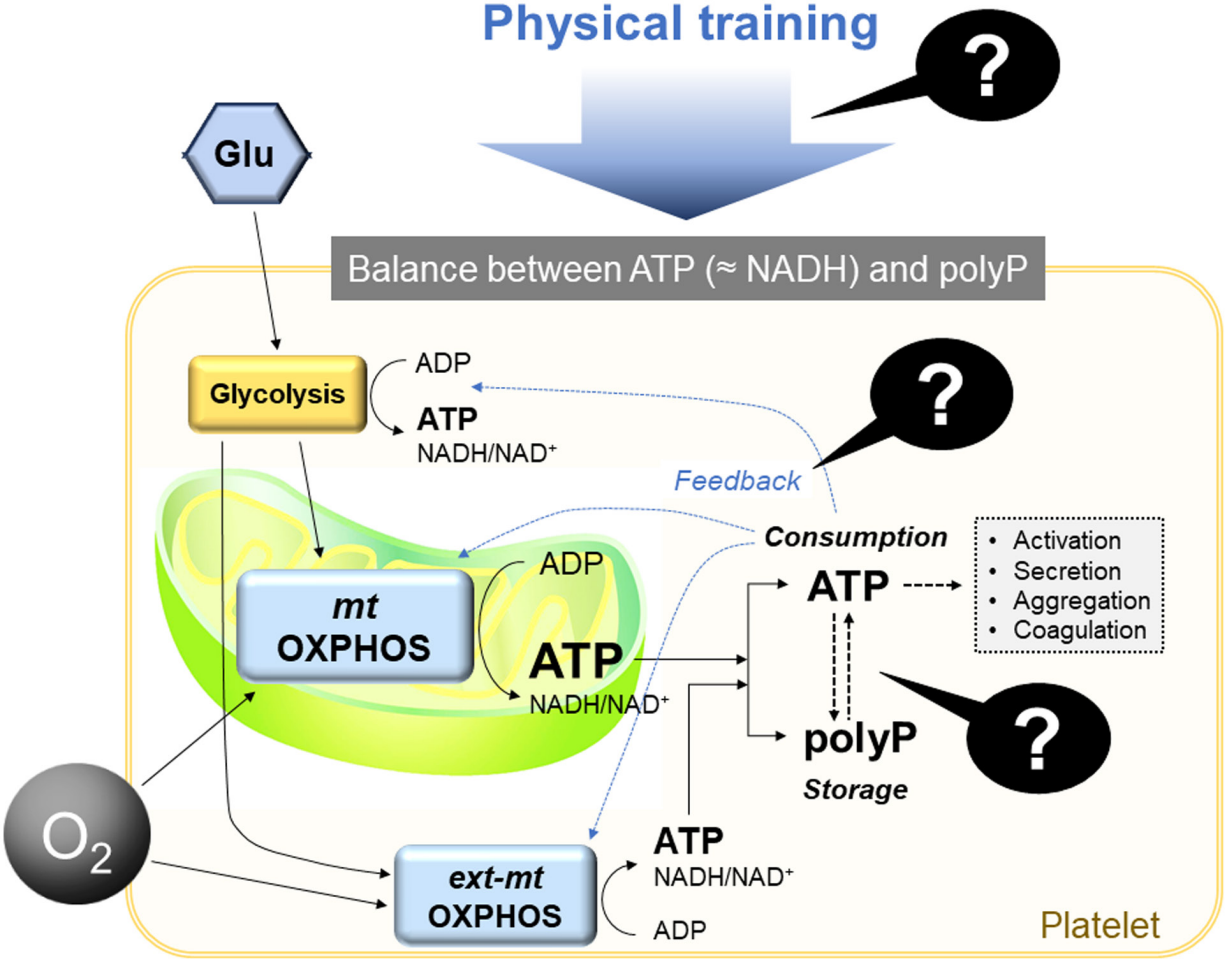
A scheme illustrating platelet energy metabolism and the objective of the present study.

In the present study, we focused on this homology (or similarity) and aimed to explore the possible differences in platelet polyP levels between non‐trained subjects and well‐trained professional athletes (Figure 1). Based on the concept of mutual conversion between ATP/ADP and polyP (Borden et al., 2021; Müller et al., 2017; Pavlov et al., 2010), we postulated that elevated polyP levels are produced and stored in the platelets of elite athletes exhibiting more active energy generation systems than non‐athlete adults. Considering this quantification, we recently simplified and optimized a fluorescence intensity quantification protocol for polyP using 4′,6‐diamidino‐2‐phenylindole (DAPI) to handle numerous samples quickly without compromising reproducibility (Uematsu et al., 2022; Watanabe et al., 2021).

## 2 MATERIALS AND METHODS

### 2.1 Study design

A cross‐sectional study was performed in two independent groups of healthy male Japanese adults (aged 19–36 years): one (non‐athlete: control) was composed of ordinary healthy adults, while the other (pro‐athlete) was composed of professional soccer players who played in the domestic professional soccer league (J. League). The inclusion criteria for the control group were as follows: healthy male young adults, who were non‐smokers, had no systemic diseases regardless of medical control, underwent no daily physical training, and agreed to provide informed consent. Exclusion criteria included acute or chronic inflammatory conditions reflected in blood cell counts and current or former thrombotic or platelet disorders. Inclusion and exclusion criteria for the pro‐athlete group were identical to that of the control group (non‐athlete), with an additional criterion of continuous daily physical training.

The study design and consent forms for all procedures (approval no. 2021‐0126) were approved by the Ethics Committee for Human Participants at the Niigata University (Niigata, Japan) and complied with the Helsinki Declaration of 1964, as revised in 2013.

### 2.2 Blood collection and preparation of platelet suspensions

Blood was collected from donors between meals in glass vacuum tubes containing ACD‐A (Vacutainer, Becton, Dickinson, and Company) using standard winged needle sets (21G) (Nipro). Whole‐blood samples were transported from hospitals to the laboratory by a parcel delivery service at ambient temperature (approximately 3–10°C). Before preparing platelet suspensions, samples were pre‐warmed for 4 h at 20–25°C to restore platelets in the resting state. Platelet pellets were prepared by the double‐spin method and gently suspended in phosphate‐buffered saline (PBS) to prepare platelet suspensions, subsequently divided into six aliquots (100 μl) as described previously. (Watanabe et al., 2021) These platelet suspensions were fixed with ThromboFix (Beckman‐Coulter) for 24 h at 4°C to stabilize cell‐surface polyP.

### 2.3 Blood cell counting

Blood cell counting was performed using an automated hematology analyzer (pocHiV‐diff, Sysmex Corporation) before centrifugation for preparation of platelet suspension and prior to determining platelet polyP levels to adjust platelet counts (Aizawa et al., 2020; Watanabe et al., 2021). In addition to cell counting, data concerning histograms of platelet volumes and the mean platelet volume (MPV) were obtained. If histograms of platelet distribution did not display a smooth curve, the samples were discarded and not subjected to polyP quantification.

### 2.4 Quantification of platelet polyP levels

Briefly, fixed platelets were centrifuged, and the resulting platelet pellets were gently suspended in Milli‐Q water. An automated hematology analyzer was used to determine blood cell count and hemoglobin (HGB) levels. Samples within the optimized range were probed with 4 μl/sample (4 μg/ml) DAPI for 30 min at room temperature (18–22°C) and directly subjected to fluorescence measurements using a fluorometer (FC‐1; Tokai Optical Co., Ltd.) with excitation and emission wavelengths of 425 and 525 nm, respectively (Uematsu et al., 2022; Watanabe et al., 2021).

### 2.5 Quantification of platelet NADH levels

Platelet NADH levels were evaluated based on the ability to reduce the tetrazolium salt WST‐8 (Dojindo Molecular Technologies, Inc.) to water‐soluble formazan. The number of non‐fixed, living platelets suspended in PBS was adjusted to a density ranging between 2.5 and 5.0 × 10^7^/100 μl, mixed with 10 μl WST‐8 solution (Dojindo Molecular Technologies, Inc.), and incubated for 1 h at room temerature (18‐23°C) under a dark environment. After centrifugation, the supernatants were analyzed using a spectrophotometer at 450 nm (SmartSpec Plus; Bio‐Rad Laboratories, Inc.), as described previously (Uematsu et al., 2022; Watanabe et al., 2021).

### 2.6 Determination of body composition

Before blood collection, the body composition of donors was determined using a bathroom weighing scale (HCS‐FS03; ECLEAR, ELECOM). This scale was installed with a unique MRI‐based program (So et al., 2012) that enables a more accurate determination of individual body fat percentages (BFP) and measured body weight, along with bioelectrical impedance. Herein, we estimated body mass index (BMI), BFP, and basal metabolic rate (BMR).

### 2.7 Statistical analysis

To compare each index between the two groups, data were expressed as box plots, and the Mann–Whitney U test was performed to confirm statistical differences in the median and spread (SigmaPlot version 14.5; Systat Software, Inc., Systat Software, Inc.). For comparison of each correlation between two indexes, Spearman's correlation analysis was performed, and correlation coefficients were calculated using SigmaPlot software. Differences were considered statistically significant at *p* < 0.05.

## 3 RESULTS

In the present study, as the pro‐athlete group was limited and could not be further expanded, non‐athlete adults of comparable age were enrolled and subjected to analyses.

**FIGURE 2:**
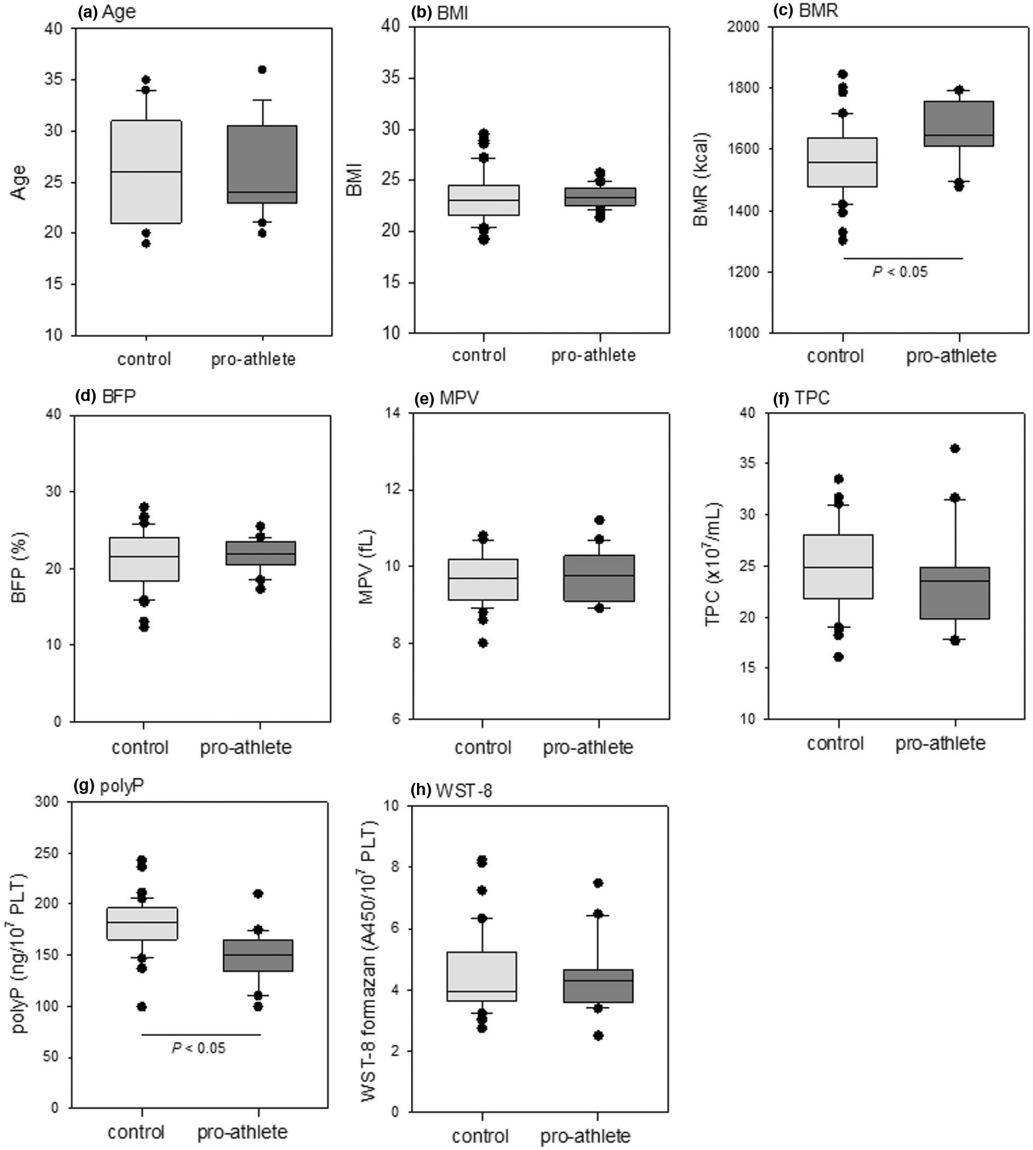
Comparisons of age (a), body composition indexes (BMI [b], BMR [c], and BFP [d]), platelet volumes (e) and counts (f), polyP levels (g), and reduced WST‐8 levels (h) between the control (non‐athlete) and pro‐athlete groups. BFP, body fat percentage; BMI, body mass index; BMR, basal metabolic rate; polyp, polyphosphate.

Figure 2 shows the comparison of age, body composition indices (BMI, BMR, and BFP), platelet volumes and counts, polyP levels, and reduced WST‐8 levels, which represent platelet NADH levels, between the control (non‐athletes) and pro‐athlete groups. As both groups were of the same age range, there were no significant differences in their age or body composition, such as BMI and BFP. Neither platelet volume nor platelet count was significantly different between the two groups. On the other hand, significant differences in functional indices such as BMR and polyP values were observed between the two groups (Figure 2c,g). The pro‐athletes exhibited higher and lower BMR and polyP values, respectively, than those in the control subjects.

**FIGURE 3:**
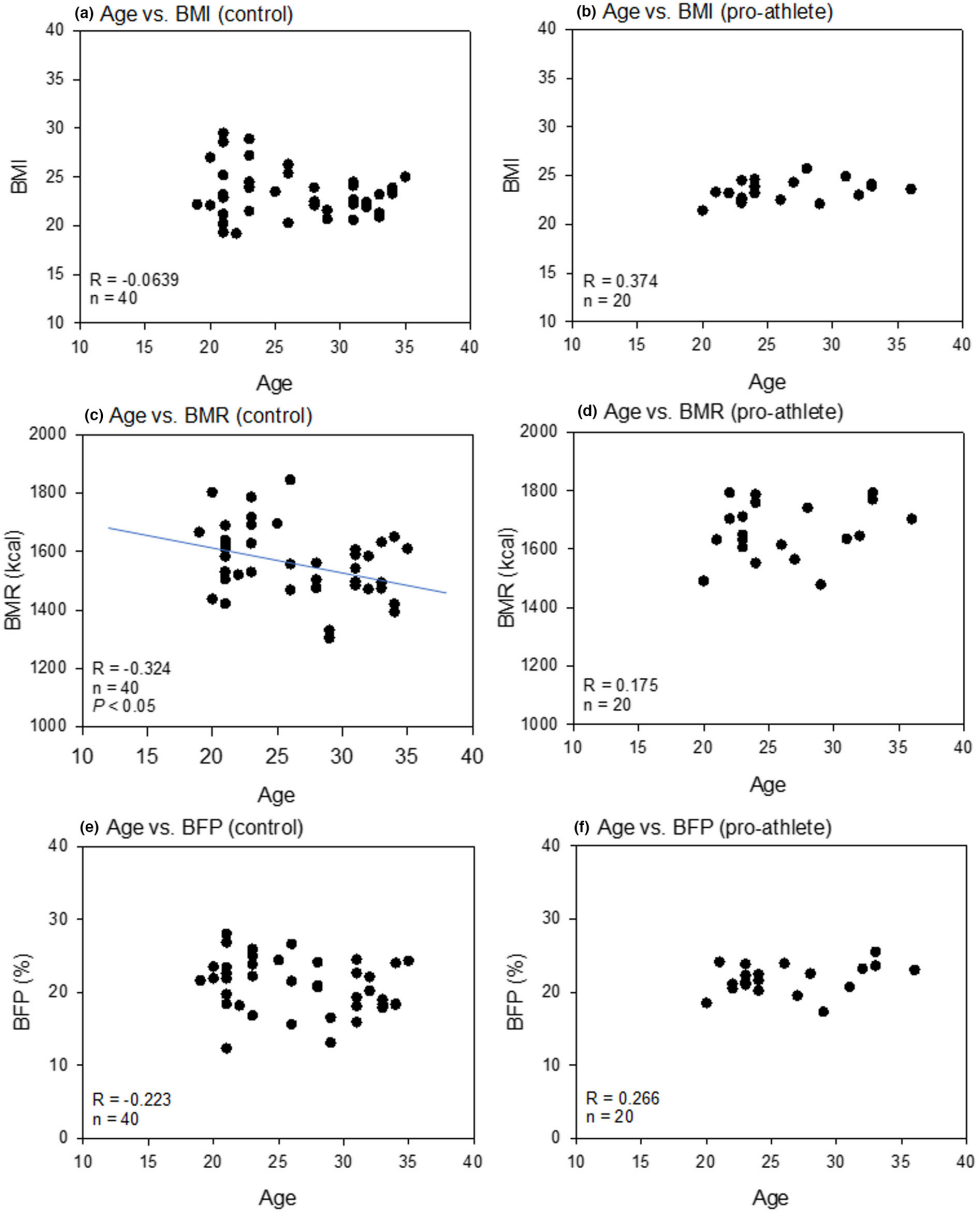
Correlations between age and body composition indexes (BMI [a, b], BMR [c, d], and BFP [e, f]) in the non‐athlete (a, c, e) and pro‐athlete (b, d, f) groups. “*R*” and “*n*” represent Spearman's correlation coefficient and sample size, respectively. BFP, body fat percentage; BMI, body mass index; BMR, basal metabolic rate.

Figure 3 shows the correlation between age and body composition indices (BMI, BMR, and BFP) in non‐athletes and pro‐athletes. We noted a weak (*R* = −0.324) but significant negative correlation between age and BMR in the control group (Figure 3c). However, there was no significant correlation between age and BMI or BFP in the control group (Figure 3a,e). Owing to the relatively narrow variances in body composition indices, significant correlations were observed in the pro‐athlete group (Figure 3b,d,f).

**FIGURE 4:**
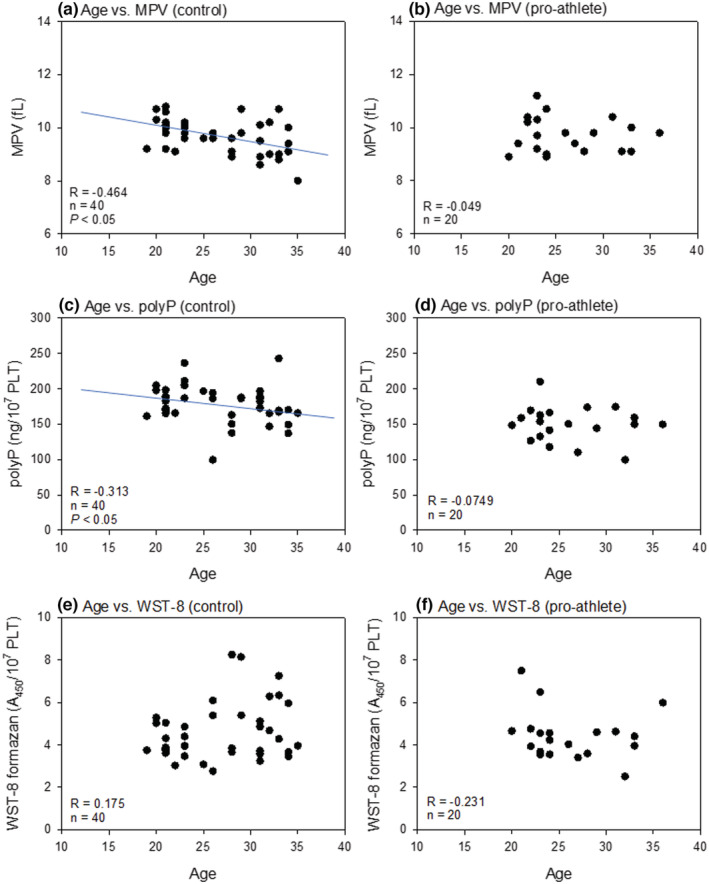
Correlations between age and platelet volumes (a, b), polyP levels (c, d), or reduced WST‐8 levels (e, f) in the non‐athlete (a, c, e) and the pro‐athlete (b, d, f) groups. polyP, polyphosphate.

Figure 4 shows the correlations between age and platelet volume, polyP levels, and reduced WST‐8 levels in nonathletes and pro‐athletes. Moderate (*R* = −0.464) and weak (*R* = −0.313), but significant negative correlations were observed between age and MPV, as well as between age and polyP levels in the control group (non‐athletes; Figure 4a,c). In the pro‐athlete group, MPV, polyP, and NADH levels were distributed at similar constant levels; therefore, no significant correlations were observed for any combination (Figure 4b,d,f).

**FIGURE 5:**
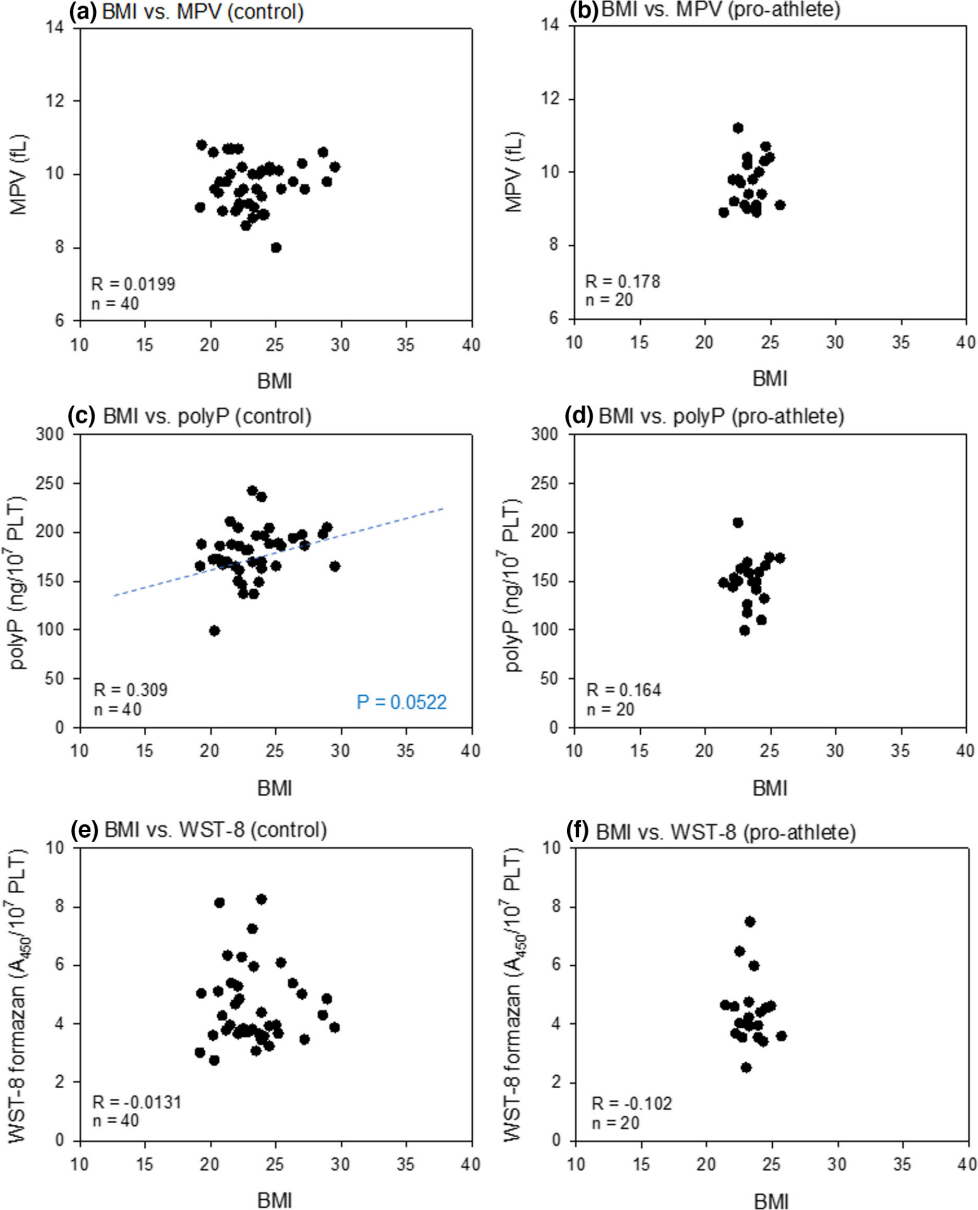
Correlations between BMI and platelet volumes (a, b), polyP levels (c, d), or reduced WST‐8 levels (e, f) in the non‐athlete (a, c, e) and pro‐athlete (b, d, f) groups. BMI, body mass index; polyP, polyphosphate.

Figure 5 shows the correlations between BMI and platelet volume, polyP levels, and reduced WST‐8 levels in non‐athletes and pro‐athletes. A weak (*R* = 0.309), but nearly significant (*p* = 0.0522), positive correlation was observed between BMI and polyP levels in the control group (non‐athletes; Figure 5c). However, very weak and non‐significant correlations were observed for other combinations in the non‐athlete group (Figure 5a,e). In contrast, the pro‐athlete group exhibited a very narrow range of BMI distribution, which appeared less appropriate for correlation evaluation (Figure 5b,d,f).

**FIGURE 6:**
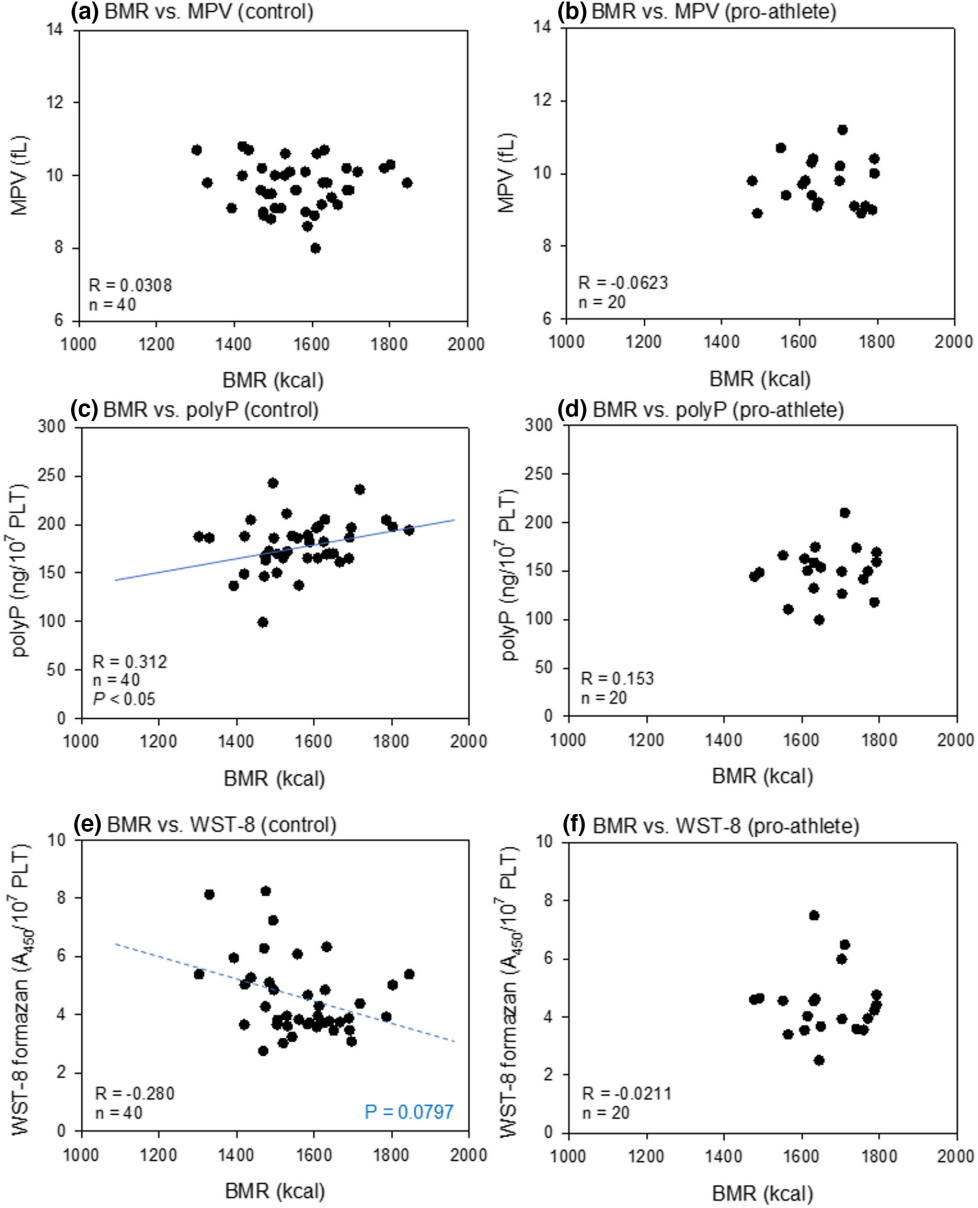
Correlations between BMR and platelet volumes (a, b), polyP levels (c, d), or reduced WST‐8 levels (e, f) in the non‐athlete (a, c, e) and pro‐athlete (b, d, f) groups. BMR, basal metabolic rate; polyP, polyphosphate.

Figure 6 shows the correlations between BMR and platelet volume, polyP levels, and reduced WST‐8 levels in nonathletes and pro‐athletes. We detected weak positive (*R* = 0.0308) and negative (*R* = −0.280) correlations between BMR and polyP, and between BMR and reduced WST‐8, respectively, in the control group (non‐athletes). In the pro‐athlete group, since the BMR values were distributed in relatively narrow ranges, no significant correlations were observed.

**FIGURE 7:**
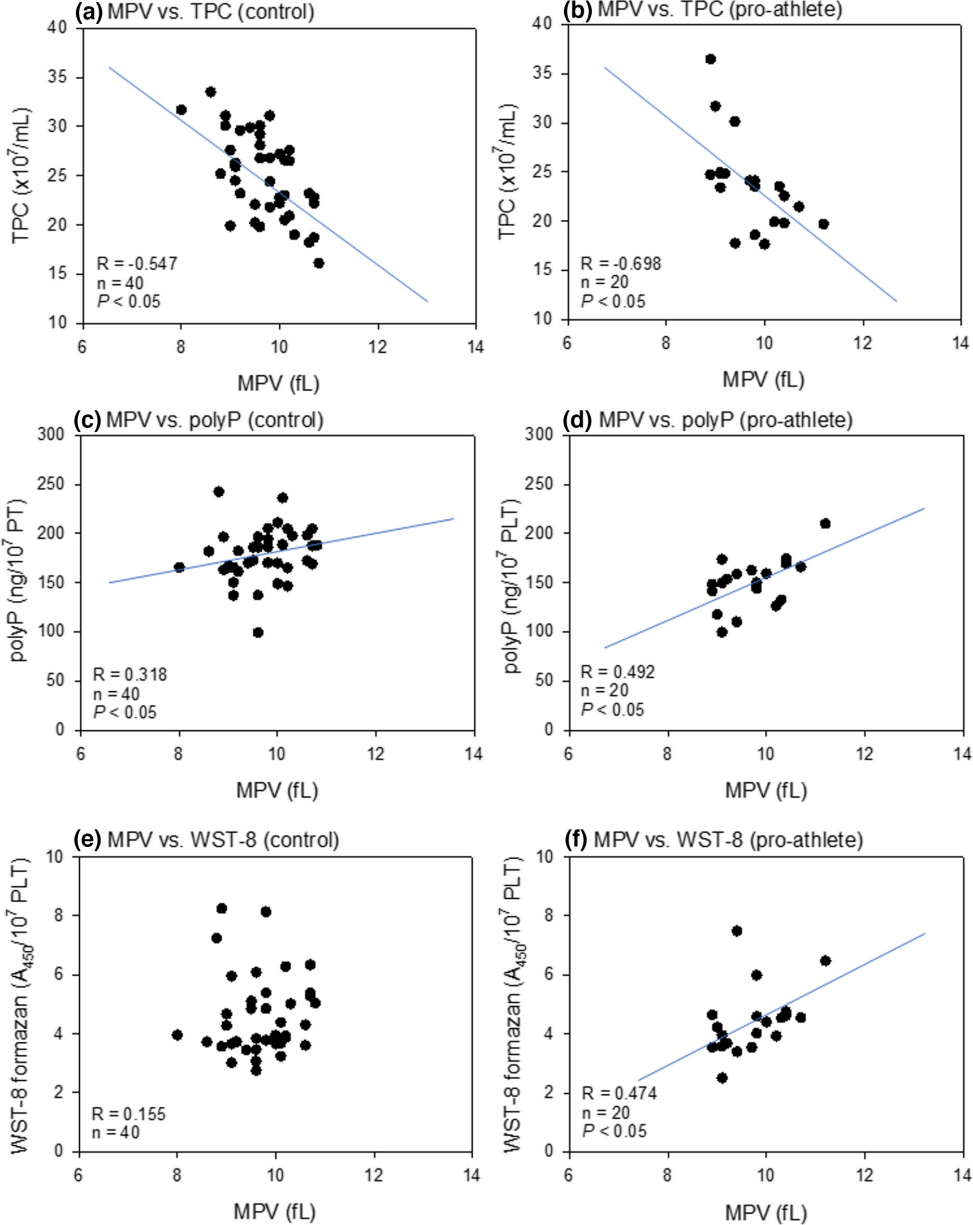
Correlations between MPV and platelet counts (a, b), polyP levels (c, d), or reduced WST‐8 levels (e, f) in the non‐athlete (a, c, e) and the pro‐athlete (b, d, f). MPV, mean platelet volume; polyP, polyphosphate.

Figure 7 shows the correlations between MPV and platelet counts, polyP levels, and reduced WST‐8 levels in non‐athletes and pro‐athletes. In both the control (non‐athlete) and pro‐athlete groups, we noted weak–moderate positive (*R* = 0.318, 0.492) and moderate negative correlations (*R* = −0.547, −0.698) between MPV and total platelet count and MPV and polyP, respectively (Figure 7a–d). Furthermore, a moderate positive correlation (*R* = 0.474) was detected between MPV and reduced WST‐8 in the pro‐athlete group.

**FIGURE 8:**
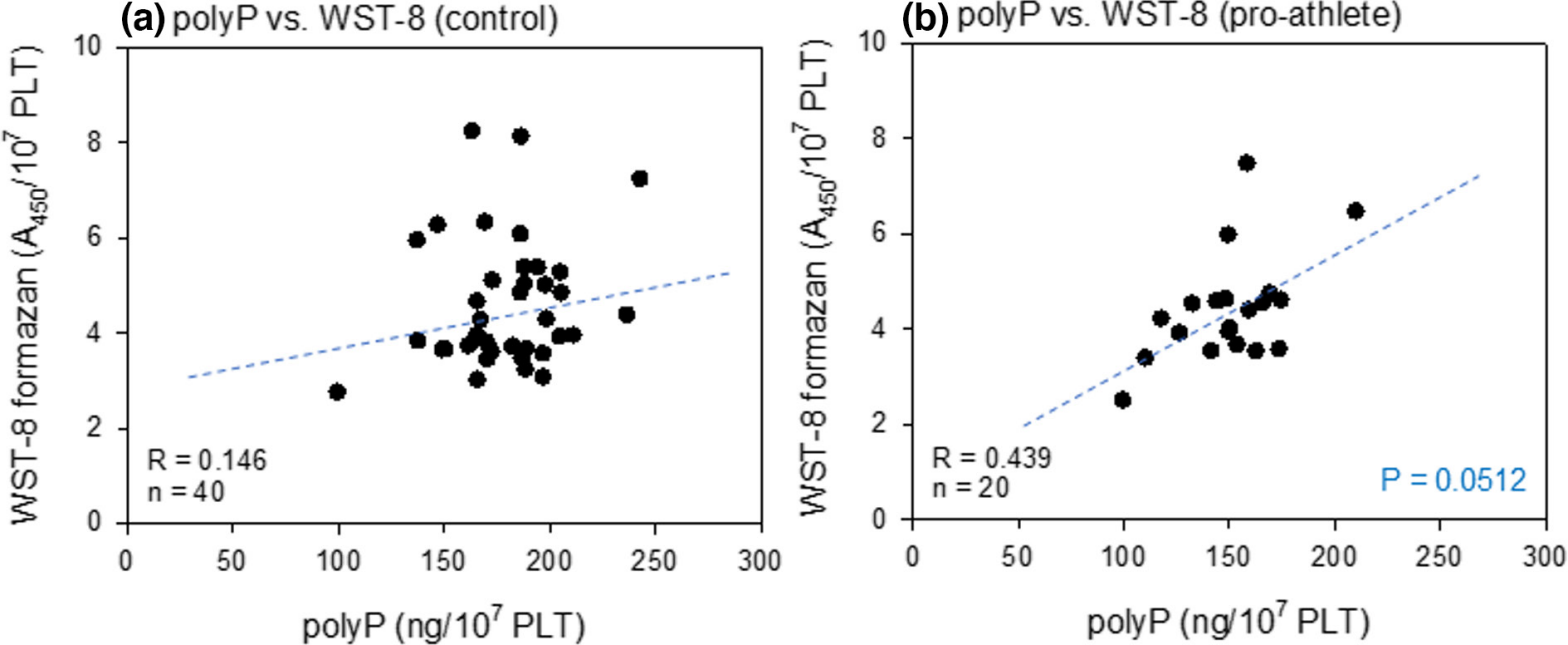
Correlations between polyP levels and reduced WST‐8 levels in the non‐athlete (a) and pro‐athlete (b) groups. polyP, polyphosphate.

Figure 8 represents the correlation between polyP and reduced WST‐8 levels in non‐athletes and pro‐athletes. We documented a moderate (*R* = 0.439), nearly‐significant (*p* = 0.0512) positive correlation between polyP and reduced WST‐8 levels only in pro‐athletes (Figure 8b).

## 4 DISCUSSION

The present study was designed and performed based on a previous report (Braganza et al., 2019). Contrary to the working hypotheses, platelet polyP levels were significantly lower in the pro‐athlete group than in the control group (non‐athletes). In addition, except for BMR, it should be noted that there were no significant differences in most body composition or platelet indexes between the two groups. Weak–moderate correlations were observed between the following comparisons: age versus BMR, age versus MPV, age versus polyP, BMI versus polyP, and BMR versus polyP in the control group. In the pro‐athlete group, most body composition indexes showed few variations and were within narrow ranges. Thus, no significant correlations were detected in any combination of body composition indexes and other parameters.

### 4.1 Assumed NADH levels and theoretically paralleled ATP levels

Accordingly, it is appropriate to discuss and explain these phenomena based on changes in intracellular energy metabolism rather than systemic body metabolism. It is well‐established that well‐scheduled appropriate physical training increases skeletal muscle strength. In addition, such training can also increase total erythrocyte volume, total hemoglobin, and cardiac output (Mairbäurl, 2013). In addition, high‐intensity training was recently shown to increase ATP production, as determined by non‐stoichiometric changes in the mitochondrial proteome, without increasing respiratory chain content in human skeletal muscle. (Granata et al., 2021) Therefore, elevated muscle performance is supported by a sufficient oxygen supply for ATP generation. Based on their concurrent dynamic changes (Protti et al., 2015), we measured tetrazolium salt (WST‐8) reduction to estimate platelet ATP levels, instead of directly measuring chemically unstable ATP levels.

Compared with that of the control group (non‐athletes), pro‐athletes showed higher ratios of assumed NADH to polyP levels. This finding implies that NADH/NAD^+^ is produced and maintained at higher levels, and/or that polyP is produced and stored at lower levels. Considering the increased oxygen supply, it is conceivable that the NADH/NAD^+^ molecule could be produced along with ATP via upregulated OXPHOS in the relatively long term, for example, after continuous daily physical training. However, in the short term, it is theoretically possible that NADH levels decrease in parallel with increased ATP production because NADH is converted to its oxidized form, NAD^+^, by providing electrons to mitochondrial OXPHOS for ATP production (Xie et al., 2020). In addition, the possibility that the activated energy production‐consumption cycle may reduce polyP storage cannot be ruled out. Additional in‐depth biochemical investigations are required to clarify this possibility.

### 4.2 Possible switching of intraplatelet energy metabolism in pro‐athletes

In the classic concept (Kilkson et al., 1984), ATP is produced mainly (~85%) by OXPHOS in resting platelets, while this system is switched to glycolysis upon activation to rapidly supply ATP to their driving system (Garcia‐Souza & Oliveira, 2014). However, recent studies have proposed the conflicting concept that both glycolysis and OXPHOS participate in energy production in resting platelets. In addition, it is speculated that, although initiated by glycolysis, OXPHOS is primarily responsible for ATP production in activated platelets (Ravera et al., 2018). In the present study, the examined platelets were considered to be in a resting state; hence, we cannot provide any insight into this controversy. Instead, we speculate that the above‐mentioned energy cycle may be switched depending on the level of physical training.

**FIGURE 9:**
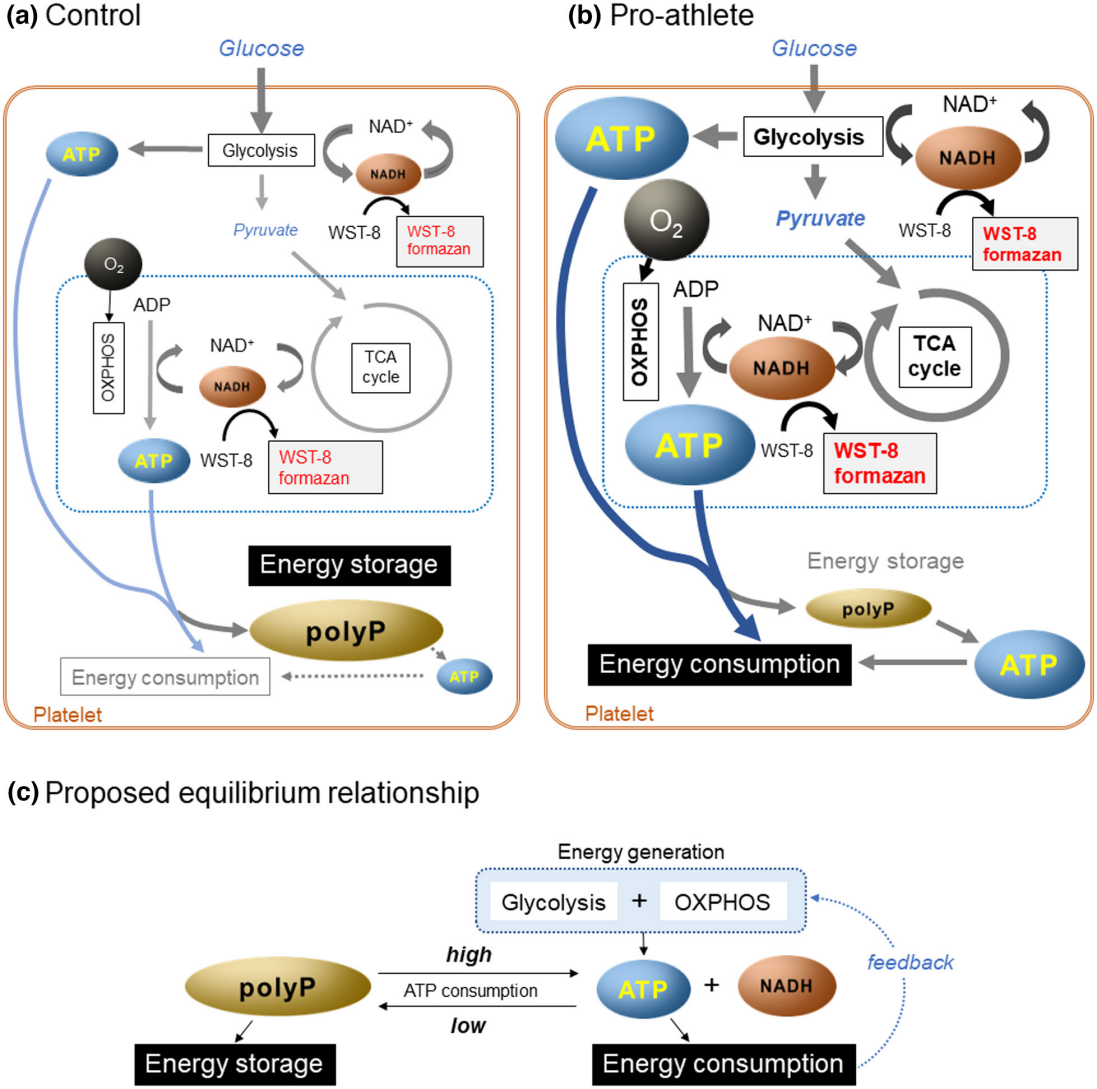
(a, b) proposed differences in platelet energy metabolism between the control (non‐athlete) (a) and the pro‐athlete (b). (c) Proposed equilibrium between ATP and polyP in platelets.

In our proposed scenario (Figure 9a,b), ATP generation is maintained at lower levels, as assessed by tetrazolium reduction, but polyP is stored as an energy source at higher levels in platelets of non‐athletes. In addition, stored polyP may not be reused frequently, and levels may increase over time, although the lifespan of platelets is limited. In contrast, the levels of ATP generation and consumption are higher in the platelets of pro‐athletes than in non‐athletes, and thus, ATP may be less used for energy storage. In addition, although a significant amount of polyP is produced, it may undergo frequent conversion to ATP in response to an urgent energy shortage.

### 4.3 Possible equilibrium between intraplatelet ATP and polyP levels

The process of polyP production in mammalian cells has not been comprehensively elucidated. However, recent studies have proposed a possible mutual conversion between ATP and polyP (Baev et al., 2020; Borden et al., 2021; Boyineni et al., 2020; McIntyre & Solesio, 2021; Müller et al., 2017, 2019; Pavlov et al., 2010). Taken together, these results suggest that the polyP level is equilibrated with the ATP level in platelets. In our proposed scenario (Figure 9c), the greater the levels of ATP present during the production‐consumption cycle, the higher the polyP levels that can be stored. However, when increased energy is required by a well‐trained male subject, such as a pro‐athlete, polyP could be recycled to continuously supply ATP (Müller et al., 2017). In contrast, even if ATP production activity is low, high levels of polyP can be stored at low ATP consumption in non‐trained subjects. Thus, we believe that appropriate physical training increases systemic body metabolism, as shown by the BMR in the present study, as well as enhances intraplatelet energy metabolism and sustains platelet polyP at low levels. Given that the platelet capacity for ATP storage is thought to differ from that of muscle storage (Hargreaves & Spriet, 2020), the possible causal relationship between physical training and platelet polyP accumulation should be further investigated at the molecular level.

### 4.4 Clinical relevance

The major limitation of the present study is the ability of the tetrazolium salt to evaluate the sum of platelet NADH levels. However, considering the proposed homology of the energy state between platelets and skeletal muscles, this cross‐sectional study introduces the possibility that polyP could be a promising, convenient biomarker for rapid monitoring of the energy state of skeletal muscle.

## 5 CONCLUSION

Despite the small sample sizes, the present study demonstrated that platelet polyP levels were significantly lower in pro‐athletes than in control subjects (non‐athlete) and moderately correlated with NADH levels. Thus, it can be suggested that platelets of pro‐athletes may exhibit elevated energy metabolic activity similar to their skeletal muscles. PolyP accumulation may be elevated in platelets with low energy metabolism, such as in non‐trained adults.

## AUTHOR CONTRIBUTIONS

T. Ushiki, T. Mochizuki, and T. Kawase conceptualized the study and provided essential resources. T. Ushiki, T. Mochizuki, K. Suzuki, M. Kamimura, H. Ishiguro, S. Watanabe, G. Omori, N. Yamamoto, T. Kawase did the collection of samples, measurements, and/or analyzed data. T. Ushiki, T. Mochizuki, and T. Kawase wrote the manuscript with comments from all authors.

## FUNDING INFORMATION

This study was financially supported by Niigata University Interdisciplinary Research (U‐go) Grant and JSPS KAKENHI (#22K11496).

## CONFLICT OF INTEREST

The authors have declared the following potential conflict of interest or source of funding: The authors' current research program is funded by the U‐go program of the Niigata University.

## ETHICS STATEMENT

The study design and consent forms for all procedures (approval no. 2021‐0126) were approved by the Ethics Committee for Human Participants at Niigata University (Niigata, Japan). This study was performed in accordance with the declaration of Helsinki's guidelines and regulations.

## References

[phy215409-bib-0001] Achbergerová, L. , & Nahálka, J. (2011). Polyphosphate—An ancient energy source and active metabolic regulator. Microbial Cell Factories, 10, 63. 21816086 10.1186/1475-2859-10-63 PMC3163519

[phy215409-bib-0002] Aibibula, M. , Naseem, K. M. , & Sturmey, R. G. (2018). Glucose metabolism and metabolic flexibility in blood platelets. Journal of Thrombosis and Haemostasis, 16, 2300–2314. 30151891 10.1111/jth.14274

[phy215409-bib-0003] Aizawa, H. , Kawabata, H. , Sato, A. , Masuki, H. , Watanabe, T. , Tsujino, T. , Isobe, K. , Nakamura, M. , Nakata, K. , & Kawase, T. (2020). A comparative study of the effects of anticoagulants on pure platelet‐rich plasma quality and potency. Biomedicine, 8, 42. 10.3390/biomedicines8030042 PMC7148468 32106422

[phy215409-bib-0004] Baev, A. Y. , Angelova, P. R. , & Abramov, A. Y. (2020). Inorganic polyphosphate is produced and hydrolyzed in F0F1‐ATP synthase of mammalian mitochondria. The Biochemical Journal, 477, 1515–1524. 32270854 10.1042/BCJ20200042 PMC7200627

[phy215409-bib-0005] Balberova, O. V. , Bykov, E. V. , Medvedev, G. V. , Zhogina, M. A. , Petrov, K. V. , Petrova, M. M. , Al‐Zamil, M. , Trefilova, V. V. , Goncharova, P. S. , & Shnayder, N. A. (2021). Candidate genes of regulation of skeletal muscle energy metabolism in athletes. Genes (Basel), 12, 1682. 34828287 10.3390/genes12111682 PMC8625318

[phy215409-bib-0006] Borden, E. A. , Furey, M. , Gattone, N. J. , Hambardikar, V. D. , Liang, X. H. , Scoma, E. R. , Abou Samra, A. , D‐Gary, L. R. , Dennis, D. J. , Fricker, D. , Garcia, C. , Jiang, Z. , Khan, S. A. , Kumarasamy, D. , Kuppala, H. , Ringrose, S. , Rosenheim, E. J. , Van Exel, K. , Vudhayagiri, H. S. , … Solesio, M. E. (2021). Is there a link between inorganic polyphosphate (polyP), mitochondria, and neurodegeneration? Pharmacological Research, 163, 105211. 33010423 10.1016/j.phrs.2020.105211 PMC7855267

[phy215409-bib-0007] Boyineni, J. , Sredni, S. T. , Margaryan, N. V. , Demirkhanyan, L. , Tye, M. , Johnson, R. , Gonzalez‐Nilo, F. , Hendrix, M. J. C. , Pavlov, E. , Soares, M. B. , Zakharian, E. , & Malchenko, S. (2020). Inorganic polyphosphate as an energy source in tumorigenesis. Oncotarget, 11, 4613–4624. 33400735 10.18632/oncotarget.27838 PMC7747861

[phy215409-bib-0008] Braganza, A. , Corey, C. G. , Santanasto, A. J. , Distefano, G. , Coen, P. M. , Glynn, N. W. , Nouraie, S. M. , Goodpaster, B. H. , Newman, A. B. , & Shiva, S. (2019). Platelet bioenergetics correlate with muscle energetics and are altered in older adults. JCI Insight, 5, e128248. 10.1172/jci.insight.128248 PMC6629251 31120438

[phy215409-bib-0009] Garcia‐Souza, L. F. , & Oliveira, M. F. (2014). Mitochondria: Biological roles in platelet physiology and pathology. The International Journal of Biochemistry & Cell Biology, 50, 156–160. 24569121 10.1016/j.biocel.2014.02.015

[phy215409-bib-0010] Granata, C. , Caruana, N. J. , Botella, J. , Jamnick, N. A. , Huynh, K. , Kuang, J. , Janssen, H. A. , Reljic, B. , Mellett, N. A. , Laskowski, A. , Stait, T. L. , Frazier, A. E. , Coughlan, M. T. , Meikle, P. J. , Thorburn, D. R. , Stroud, D. A. , & Bishop, D. J. (2021). High‐intensity training induces non‐stoichiometric changes in the mitochondrial proteome of human skeletal muscle without reorganisation of respiratory chain content. Nature Communications, 12, 7056. 10.1038/s41467-021-27153-3 PMC8642543 34862379

[phy215409-bib-0011] Greenhaff, P. L. , Campbell‐O'Sullivan, S. P. , Constantin‐Teodosiu, D. , Poucher, S. M. , Roberts, P. A. , & Timmons, J. A. (2002). An acetyl group deficit limits mitochondrial ATP production at the onset of exercise. Biochemical Society Transactions, 30, 275–280. 12023864 10.1042/

[phy215409-bib-0012] Hambardikar, V. , Guitart‐Mampel, M. , Scoma, E. R. , Urquiza, P. , Nagana, G. G. A. , Raftery, D. , Collins, J. A. , & Solesio, M. E. (2022). Enzymatic depletion of mitochondrial inorganic polyphosphate (polyP) increases the generation of reactive oxygen species (ROS) and the activity of the pentose phosphate pathway (PPP) in mammalian cells. Antioxidants, 11, 685. 35453370 10.3390/antiox11040685 PMC9029763

[phy215409-bib-0013] Hargreaves, M. , & Spriet, L. L. (2020). Skeletal muscle energy metabolism during exercise. Nature Metabolism, 2, 817–828. 10.1038/s42255-020-0251-4 32747792

[phy215409-bib-0014] Hoppel, F. , Calabria, E. , Pesta, D. H. , Kantner‐Rumplmair, W. , Gnaiger, E. , & Burtscher, M. (2021). Effects of ultramarathon running on mitochondrial function of platelets and oxidative stress parameters: A pilot study. Frontiers in Physiology, 12, 632664. 33679442 10.3389/fphys.2021.632664 PMC7935014

[phy215409-bib-0015] Kilkson, H. , Holme, S. , & Murphy, S. (1984). Platelet metabolism during storage of platelet concentrates at 22 degrees C. Blood, 64, 406–414. 6430365

[phy215409-bib-0016] Knuiman, P. , Hopman, M. T. , & Mensink, M. (2015). Glycogen availability and skeletal muscle adaptations with endurance and resistance exercise. Nutrition & Metabolism (London), 12, 59. 10.1186/s12986-015-0055-9 PMC4687103 26697098

[phy215409-bib-0017] Mairbäurl, H. (2013). Red blood cells in sports: Effects of exercise and training on oxygen supply by red blood cells. Frontiers in Physiology, 4, 332. 24273518 10.3389/fphys.2013.00332 PMC3824146

[phy215409-bib-0018] McIntyre, B. , & Solesio, M. E. (2021). Mitochondrial inorganic polyphosphate (polyP): The missing link of mammalian bioenergetics. Neural Regeneration Research, 16, 2227–2228. 33818504 10.4103/1673-5374.310687 PMC8354130

[phy215409-bib-0019] Melchinger, H. , Jain, K. , Tyagi, T. , & Hwa, J. (2019). Role of platelet mitochondria: Life in a nucleus‐free zone. Frontiers in Cardiovascular Medicine, 6, 153. 31737646 10.3389/fcvm.2019.00153 PMC6828734

[phy215409-bib-0020] Müller, W. E. G. , Schröder, H. C. , & Wang, X. (2019). Inorganic polyphosphates as storage for and generator of metabolic energy in the extracellular matrix. Chemical Reviews, 119, 12337–12374. 31738523 10.1021/acs.chemrev.9b00460 PMC6935868

[phy215409-bib-0021] Müller, W. E. G. , Wang, S. , Neufurth, M. , Kokkinopoulou, M. , Feng, Q. , Schröder, H. C. , & Wang, X. (2017). Polyphosphate as a donor of high‐energy phosphate for the synthesis of ADP and ATP. Journal of Cell Science, 130, 2747–2756. 28687622 10.1242/jcs.204941

[phy215409-bib-0022] Pavlov, E. , Aschar‐Sobbi, R. , Campanella, M. , Turner, R. J. , Gómez‐García, M. R. , & Abramov, A. Y. (2010). Inorganic polyphosphate and energy metabolism in mammalian cells. The Journal of Biological Chemistry, 285, 9420–9428. 20124409 10.1074/jbc.M109.013011 PMC2843191

[phy215409-bib-0023] Protti, A. , Fortunato, F. , Artoni, A. , Lecchi, A. , Motta, G. , Mistraletti, G. , Novembrino, C. , Comi, G. P. , & Gattinoni, L. (2015). Platelet mitochondrial dysfunction in critically ill patients: Comparison between sepsis and cardiogenic shock. Critical Care, 19, 39. 25757508 10.1186/s13054-015-0762-7 PMC4338849

[phy215409-bib-0024] Ravera, S. , & Panfoli, I. (2019). Platelet aerobic metabolism: New perspectives. Journal of Unexplored Medical Data, 4, 7.

[phy215409-bib-0025] Ravera, S. , Signorello, M. G. , Bartolucci, M. , Ferrando, S. , Manni, L. , Caicci, F. , Calzia, D. , Panfoli, I. , Morelli, A. , & Leoncini, G. (2018). Extramitochondrial energy production in platelets. Biology of the Cell, 110, 97–108. 29537672 10.1111/boc.201700025

[phy215409-bib-0026] Simbulan‐Rosenthal, C. , Carney, B. , Gaur, A. , Moghe, M. , Crooke, E. , Moffatt, L. , Shupp, J. , & Rosenthal, D. (2019). Inorganic polyphosphates are important for cell survival and motility of human skin keratinocytes and play a role in wound healing. In D. Churchill , M. Sikirić , B. Čolović , & H. Milhofer (Eds.), Contemporary topics about phosphorus in biology and Materials. IntechOpen.

[phy215409-bib-0027] So, R. , Sasai, H. , Matsuo, T. , Tsujimoto, T. , Eto, M. , Saotome, K. , & Tanaka, K. (2012). Multiple‐slice magnetic resonance imaging can detect visceral adipose tissue reduction more accurately than single‐slice imaging. European Journal of Clinical Nutrition, 66, 1351–1355. 23093345 10.1038/ejcn.2012.147

[phy215409-bib-0028] Tyrrell, D. J. , Bharadwaj, M. S. , Jorgensen, M. J. , Register, T. C. , & Molina, A. J. (2016). Blood cell respirometry is associated with skeletal and cardiac muscle bioenergetics: Implications for a minimally invasive biomarker of mitochondrial health. Redox Biology, 10, 65–77. 27693859 10.1016/j.redox.2016.09.009 PMC5045569

[phy215409-bib-0029] Tyrrell, D. J. , Bharadwaj, M. S. , Jorgensen, M. J. , Register, T. C. , Shively, C. , Andrews, R. N. , Neth, B. , Keene, C. D. , Mintz, A. , Craft, S. , & Molina, A. J. A. (2017). Blood‐based bioenergetic profiling reflects differences in brain bioenergetics and metabolism. Oxidative Medicine and Cellular Longevity, 2017, 7317251–7317259. 29098063 10.1155/2017/7317251 PMC5643153

[phy215409-bib-0030] Uematsu, T. , Sato, A. , Aizawa, H. , Tsujino, T. , Watanabe, T. , Isobe, K. , Kawabata, H. , Kitamura, Y. , Tanaka, T. , & Kawase, T. (2022). Effects of SARS‐CoV‐2 mRNA vaccines on platelet polyphosphate levels and inflammation: A pilot study. Biomedical Reports, 16, 21. 35251608 10.3892/br.2022.1504 PMC8850965

[phy215409-bib-0031] Watanabe, T. , Kitamura, Y. , Aizawa, H. , Masuki, H. , Tsujino, T. , Sato, A. , Kawabata, H. , Isobe, K. , Nakata, K. , & Kawase, T. (2021). Fluorometric quantification of human platelet polyphosphate using 4′,6‐diamidine‐2‐phenylindole dihydrochloride: Applications in the Japanese population. International Journal of Molecular Sciences, 22, 7257. 34298874 10.3390/ijms22147257 PMC8307652

[phy215409-bib-0032] Winnica, D. , Corey, C. , Mullett, S. , Reynolds, M. , Hill, G. , Wendell, S. , Que, L. , Holguin, F. , & Shiva, S. (2019). Bioenergetic differences in the airway epithelium of lean versus obese asthmatics are driven by nitric oxide and reflected in circulating platelets. Antioxidants & Redox Signaling, 31, 673–686. 30608004 10.1089/ars.2018.7627 PMC6708272

[phy215409-bib-0033] Xie, N. , Zhang, L. , Gao, W. , Huang, C. , Huber, P. E. , Zhou, X. , Li, C. , Shen, G. , & Zou, B. (2020). NAD+ metabolism: Pathophysiologic mechanisms and therapeutic potential. Signal Transduction and Targeted Therapy, 5, 227. 33028824 10.1038/s41392-020-00311-7 PMC7539288

